# Supplementary material for: Elevated LSD1 and SNAIL Expression Indicate Poor Prognosis in Hypopharynx Carcinoma
Source: Int J Mol Sci. 2022 May 3;23(9):5075. doi: 10.3390/ijms23095075 (PMC9100259; doi:10.3390/ijms23095075)

Table S1. Characteristics of the cohort. data not available (d.n.a.), Primary Tumor (PT), Lymph node metastasis (LN), Distant metastasis (DM), Local recurrence (LR), Cancer of unknown primary (CUP), positive (pos.), negative (neg.), without (w/o), progression (prog), overall survival (OS), Progrssion-free survival (PFS)

		LSD1 staining n=	SNAIL staining n=
**Patients analyzed**		339	339
	d.n.a.	5	7
**Tissue Type**	PT	334	332
	LN	163	165
	DM	22	21
	LR	59	57
**Sub-Site**	Hypopharynx	46	46
	Larynx	98	95
	Oral Cavity	76	76
	Oropharynx	103	104
	CUP / d.n.a.	11	11
**Sex**	male	259	258
	female	74	73
	d.n.a.	1	1
**Age**	≤ 61	54	52
	> 61	178	178
	d.n.a.	2	2
**T-Stage**	T1	68	69
	T2	98	98
	T3	96	96
	T4	70	67
	d.n.a.	2	2
**N-Stage**	N0	147	146
	N1	51	51
	N2	83	83
	N3	50	50
	d.n.a.	3	3
**M-Stage**	M0	317	315
	M1	14	14
	d.n.a	3	3
**UICC**	I	66	69
	II	59	57
	II	59	59
	IV	149	146
	d.n.a.	1	1
**p16**	pos.	85	88
	neg.	249	244
	d.n.a.	0	0
**Alcohol cons.**	Yes	139	139
	No	183	180
	d.n.a.	2	3
**Nicotine cons.**	Yes	281	279
	No	36	35
	d.n.a.	17	18
**5 y. PFS**	dead or prog.	148	147
	alive w/o prog	178	177
	d.n.a.	8	8
**5 y. OS**	dead	119	117
	alive	207	107
	d.n.a.	8	8

Table S2. Univariate and multivariate Cox regression for PFS in primary hypopharyngeal HNSCC. Survival analysis of 5-year PFS in primary hypopharyngeal head and neck squamous cell carcinoma (HNSCC) using the Cox proportional hazards regression model. Only tumor and patient characteristics showing a significant correlation with 5-year PFS in the univariate analysis were included in the multivariate analysis. Characteristics and corresponding p-values showing a significant correlation in the multivariate analysis are bold.

**Variable**	**Category**	**Univariate HR (95% -Konf. Int., p=)**	**Multivariate HR (95%- conf. int., p=)**
**LSD1-Expression**	LSD1 low<br>LSD high	2.53<br>(1.05-6.09, p=0.038)	3.72<br>(1.24-10.94, **p=0.028**)
T- Stage	T1,2,3<br>T4	3.25<br>(1.29-8.21, p=0.013)	1.03<br>(0.35-3.01, p=0.924)
**N-Stage**	N0,1<br>N2,3	4.18<br>(1.53-11.43, p=0.005)	0.15<br>(0.02-1.02, p=0.052)
M-Stage	M0<br>M1	4.21<br>(0.53-33.28, p=0.173)	
**UICC-Stage**	UICC I,II,III<br>UICC IV	7.37<br>(2.16-25.15, p=0.001)	44.56<br>(4.18-475.45, **p=0.002**)
Sex	Male<br>Female	0.39<br>(0.09-1.66, p=0.202)	
Age	≤ 61 y.<br>> 61 y.	0.97<br>(0.42-2.22, p=0.943)	
p16	Positive<br>Negative	2.76<br>(0.37-20.51, p=0.321)	
Alcohol	Yes<br>No	0.71<br>(0.28-1.75, p=0.453)	
Nicotine	Yes<br>No	2.63<br>(0.61-11.41, p=0.197)	

Table S3. Univariate and multivariate Cox regression for in primary HNSCC. 5-year OS analysis using the Cox proportional hazards regression model. Multivariate analysis was performed with all tumor and patient characteristics showing a significant correlation with patient survival in the univariate analysis. Characteristics and corresponding p-values showing a significant correlation in the multivariate analysis are bold.

**Variable**	**Category**	**Univariate HR (95% -Konf. Int., p=)**	**Multivariate HR (95%- conf. int., p=)**
**SNAIL-Expression**	SNAIL low<br>SNAIL high	1.45<br>(1.01-2.10, p=0.046)	1.57<br>(1.07-2.31, **p=0.021**)
**T- Stage**	T1,2<br>T3,4	2.14<br>(1.47-3.11, p<0,001)	1.79<br>(1.08-2.99**, p=0.025**)
N-Stage	N0<br>N1,2,3	1.67<br>(1.14-2.44, p=0.009)	1.49<br>(0.98-2.36, p=0.088)
**M-Stage**	M0<br>M1	5.15<br>(2.74-9.67, p<0.001)	5.15<br>(2.64-10.03**, p<0.001**)
UICC-Stage	UICC I,II<br>UICC III,IV	2.32<br>(1.52-3.53, p<0,001)	1.19<br>(0.61-2.34, p=0.606)
Sex	Male<br>Female	0.80<br>(0.51-1.27, p=0.345)	
Age	≤ 61 y.<br>> 61 y.	1.25<br>(0.86-1.81, p=0.236)	
p16	Positive<br>Negative	1.93<br>(1.20-3.13, p=0.007)	1.70<br>(0.96-3.01, p=0.071)
Alcohol	Yes<br>No	0.57<br>(0.40-0.84, p=0.004)	0.74<br>(0.50-1.09, p=0.125)
Nicotine	Yes<br>No	0.60<br>(0.29-1.22, p=0.158)	

Table S4. Univariate and multivariate cox regression of PFS in primary HNSCC. Survival analysis of 5-year PFS in primary HNSCC using the Cox proportional hazards regression model. All tumor and patient characteristics showing a significant correlation with 5-year survival in the univariate analysis were included in the multivariate analysis. Characteristics and corresponding p-values showing a significant correlation in the multivariate analysis are bold.

Variable	Category	Univariate HR<br>(95% -Konf. Int., p=)	Multivariate HR<br>(95%- conf. int., p=)
**SNAIL-Expression**	SNAIL low<br>SNAIL high	1.63<br>(1.17-2.26, p=0.004)	1.82<br>(1.29-2.57, **p=0.001**)
**T- Stage**	T1,2<br>T3,4	2.10<br>(1.51-2.94, p<0.001)	1.79<br>(1.12-2.85, **p=0.014**)
N-Stage	N0<br>N1,2,3	1.40<br>(1.00-1.95, p=0.051)	
**M-Stage**	M0<br>M1	3.41<br>(1.83-6.32, p<0.001)	3.45<br>(1.80-6.63**, p<0.001**)
UICC-Stage	UICC I,II<br>UICC III,IV	2.19<br>(1.52-3.16, p<0.001)	1.38<br>(0.81-2.34, p=0.238)
Sex	Male<br>Female	0.84<br>(0.56-1.26, p=0.398)	
Age	≤ 61 y.<br>> 61 y.	1.22<br>(0.88-1.69, p=0.241)	
**p16**	Positive<br>Negative	2.20<br>(1.42-3.41, p<0.001)	1.79<br>(1.10-2.90**, p=0.018**)
Alcohol	Yes<br>No	0.63<br>(0.45-0.87, p=0.006)	0.81<br>(0.57-1.14, p=0.224)
Nicotine	Yes<br>No	0.76<br>(0.43-1.35, p=0.354)	

Figure S1. Examplary HE stained TMA Cores. Examplary Tissue Micro Array (TMA) cores of the cohort stained with standard hematoxylin and eosin (HE) staining.

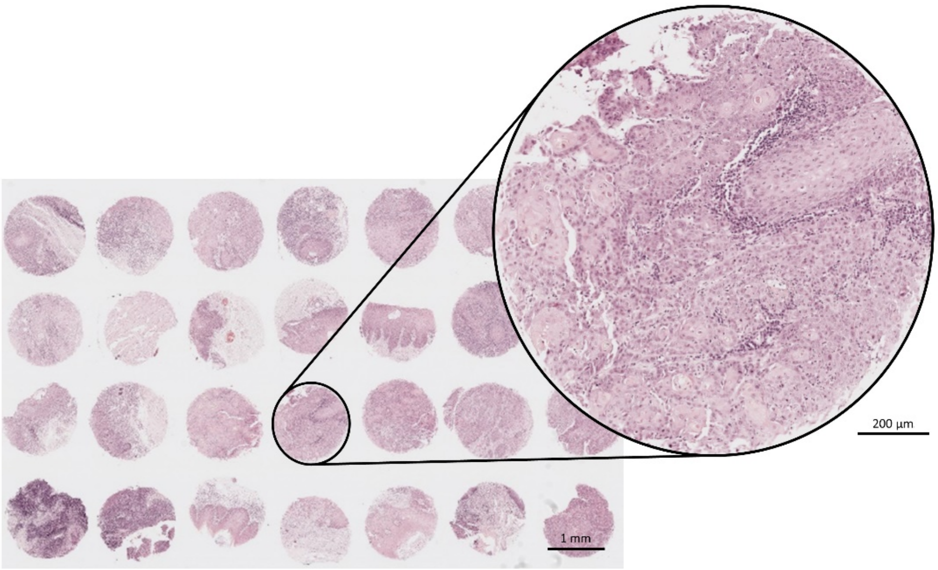

Supplement: Supplementary file 1 [file ijms-23-05075-s001.zip › ijms-1713730-supplementary.pdf]
